# Effects of short-time exposure of surface pre-reacted glass-ionomer eluate on dental microcosm biofilm

**DOI:** 10.1038/s41598-020-71363-6

**Published:** 2020-09-02

**Authors:** Hyo-Jung Kim, Mu-Yeol Cho, Eun-Song Lee, Hoi In Jung, Baek-Il Kim

**Affiliations:** 1 Department of Preventive Dentistry & Public Oral Health, Yonsei University College of Dentistry, 50-1 Yonsei-ro, Seodaemun-Gu, Seoul, 03722 Republic of Korea; 2 BK 21 PLUS Project, Yonsei University College of Dentistry, Seoul, Korea

**Keywords:** Antimicrobials, Bacteria, Biofilms

## Abstract

This study evaluated the antibacterial effects of short-time exposure of surface pre-reacted glass-ionomer (S-PRG) eluate on oral microcosm biofilm. Biofilms were treated with an S-PRG eluate at different concentrations (25%, 50%, and 100%), distilled water (DW), and 0.1% chlorhexidine (CHX) twice a day for 5 min repeatedly. After 7 days, the total and aciduric bacterial counts and biofilm dry weights were measured. An image analysis program calculated the red/green (R/G) ratios in the biofilm autofluorescence images. Microscopic analyses quantified the biofilm thickness and live/dead cell ratio and determined morphological changes in the biofilm. Bacterial counts and dry weights were not significantly different in the DW group for all S-PRG eluate concentrations. An increasing trend in the R/G ratio for 7 days biofilm treatment was observed for the S-PRG eluate and the DW groups. Furthermore, the live/dead cell ratios in the biofilm and the biofilm thickness of the S-PRG eluate groups were similar to those of the DW group. The bacteria morphology inside the biofilm changed only in the CHX group. Short-time S-PRG eluate treatment showed no significant antibacterial and antibiofilm effects. These results indicated that limited biofilm formation inhibition can be obtained by using only the S-PRG eluate.

## Introduction

Dental caries is a major oral disorder caused by the pathogenic bacteria inhabiting complex microbial communities known as biofilms^1^. Intraoral biofilms increase the resistance against planktonic bacteria and make it difficult for foreign substances to penetrate the extracellular matrix that forms a thick physical layer via an acquired pellicle stage^2^. Physical interventions including toothbrushing and the use of dental chemicals containing effective antibacterial components have been proposed as self-management methods for removing these oral biofilms^3^. Various antibacterial active ingredients are currently under development, and numerous studies have evaluated their effectiveness in preventing dental caries by examining the antibacterial and anticariogenic effects.

Various recent studies have reported the remineralization and antibacterial activities of dental restorative materials constructed using a pre-reacted glass-ionomer (PRG) technology in combination with polymers^4^. Surface pre-reacted glass-ionomer (S-PRG) filler is a material treated with a siliceous hydrogel that is formed via an acid–base reaction between fluoroboroaluminosilicate glass and polyacrylic acid. S-PRG filler is harmless to the human body and contains six ions: Al^3+^, BO_3_^3−^, Na^+^, SiO_3_^2-^, Sr^2+^, and F^–^^5^. As S-PRG fillers containing these ions have been developed as components of dental restorative materials, these have been extensively investigated in studies using resin or varnish-type preparations^6,7^. However, it is not feasible to regularly use such professional dental materials compared to self-care materials such as mouthwash. Furthermore, oral biofilms also have low accessibility.

Therefore, in recent years, in addition to the application of S-PRG as a restorative material, the effects of S-PRG in an eluate form have attracted significant attention. S-PRG eluate has been reported to inhibit the formation^8^ and adhesion^9^ of *Streptococcus mutans* in single-species biofilms. Iwamatsu‐Kobayashi et al.^10^ reported that the S-PRG eluate prevented the destruction of tissue in periodontal disease through anti-inflammatory effects. Most of the studies examining the antibacterial and antibiofilm effects on oral bacteria using an S-PRG eluate were performed only on planktonic bacteria or single-species biofilms^5,9^. According to a recent perspective on biofilm control, the determination of the efficacy of new antibacterial substances requires the measurement of the changes in the pathogenicity of the biofilm using a multispecies microcosm biofilm model that reflects a realistic oral environment, rather than simply assessing the viability against a single bacteria or a single-species biofilm.

Suzuki et al.^11^ reported that an S-PRG eluate inhibited the formation of new biofilms and disrupted mature biofilms, but this study only employed a microbial viability assay to examine the effects on the biofilm formation. In addition, most of the prior studies that reported the antimicrobial or antibiofilm effects using an S-PRG eluate formed a biofilm in a culture medium containing the S-PRG eluate. Therefore, this eluate was in contact with bacteria for more than 24 h^7,8,11^. In the present study, the antimicrobial and antibiofilm effects of the S-PRG eluate were evaluated by using a short-time exposure (5 min) of various concentrations of the S-PRG eluate on the microcosm biofilm by simulating the conditions in which mouthwash preparations are used in the actual oral cavity (e.g., with daily application, twice a day). The null hypothesis is that the S-PRG eluate has no inhibitory effect on the formation of the dental microcosm biofilms and bacteria in the biofilms.

## Results

### Inhibitory effects of S-PRG eluate on dental microcosm biofilm

All S-PRG eluate groups exhibited lower values for all parameters such as total viable count, aciduric viable count, and dry weight, compared to those of distilled water (DW) used as a negative control, but showed no significant differences (Table 1). Additionally, no statistically significant differences were observed between the values of these parameters for the S-PRG eluate groups regardless of the concentration. Contrarily, significantly lower values for these parameters were observed for 0.1% CHX, compared to those for the negative control and S-PRG groups (25, 50, and 100% S-PRG eluates; *P* < 0.001).

**Table 1:** Inhibitory effects of different treatment solutions on dental microcosm biofilms.

Treatment	Bacterial counts		Dry weight (mg/ml)
	Total (log_10_ CFUs)	Aciduric (log_10_ CFUs)	
Distilled water	8.08 (7.95–8.66)^a^	7.36 (6.91–7.68)^a^	1.30 (1.00–1.60)^a^
25% S-PRG	7.91 (7.70–8.05)^a^	7.11 (6.42–7.61)^a^	1.30 (1.05–1.40)^a^
50% S-PRG	7.63 (7.11–7.98)^a^	6.89 (6.63–7.31)^a^	1.00 (0.90–1.15)^a^
100% S-PRG	7.82 (7.48–7.94)^a^	6.94 (6.53–7.70)^a^	1.00 (0.95–1.20)^a^
0.1% CHX	6.68 (6.41–6.94)^b^	< DL	0.40 (0.40–0.55)^b^

Data represent the median (Q25–Q75) values.

*CHX* chlorhexidine. *CFUs* colony-forming units, *DL* detection limit.

Superscripts within the same column indicate significant intergroup differences (Mann–Whitney, *p* < 0.005).

### Red fluorescence intensity of biofilm

**Figure 1:**
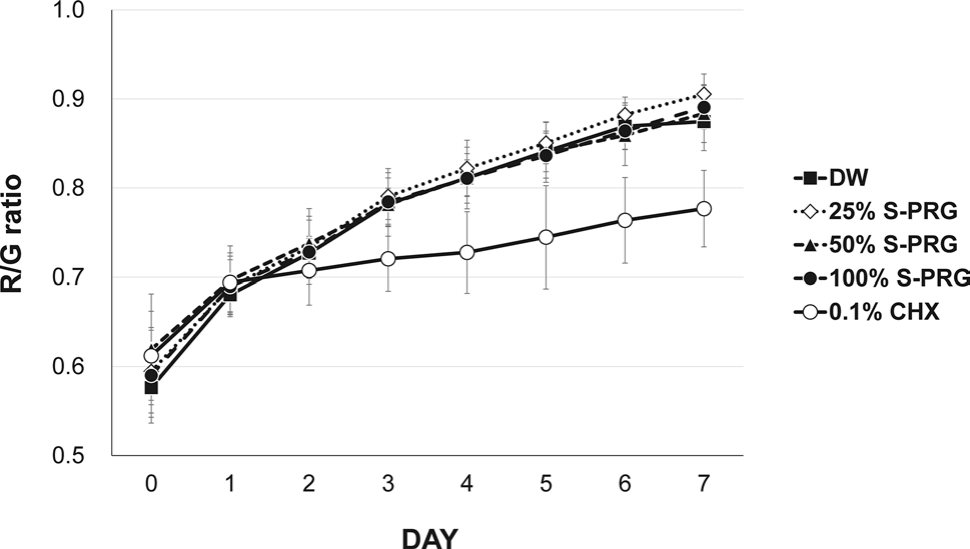
Variation in red fluorescence intensity (quantified as red/green ratios) of the microcosm biofilm grown with different experimental solutions according to the maturation time (n = 13).

Based on the red fluorescence data of the biofilm formed with the treatment of S-PRG for 7 days, the red/green (R/G) values of all groups gradually increased with maturation time (Fig. 1). No significant differences were observed in the R/G values between the DW control group and S-PRG eluate groups at all concentrations. Contrarily, the positive control, 0.1% CHX group, showed a significantly lower R/G value compared to those of the other groups from three days after inoculation (*P* < 0.001).

### Morphological characteristics of bacterial growth in the biofilm

**Figure 2:**
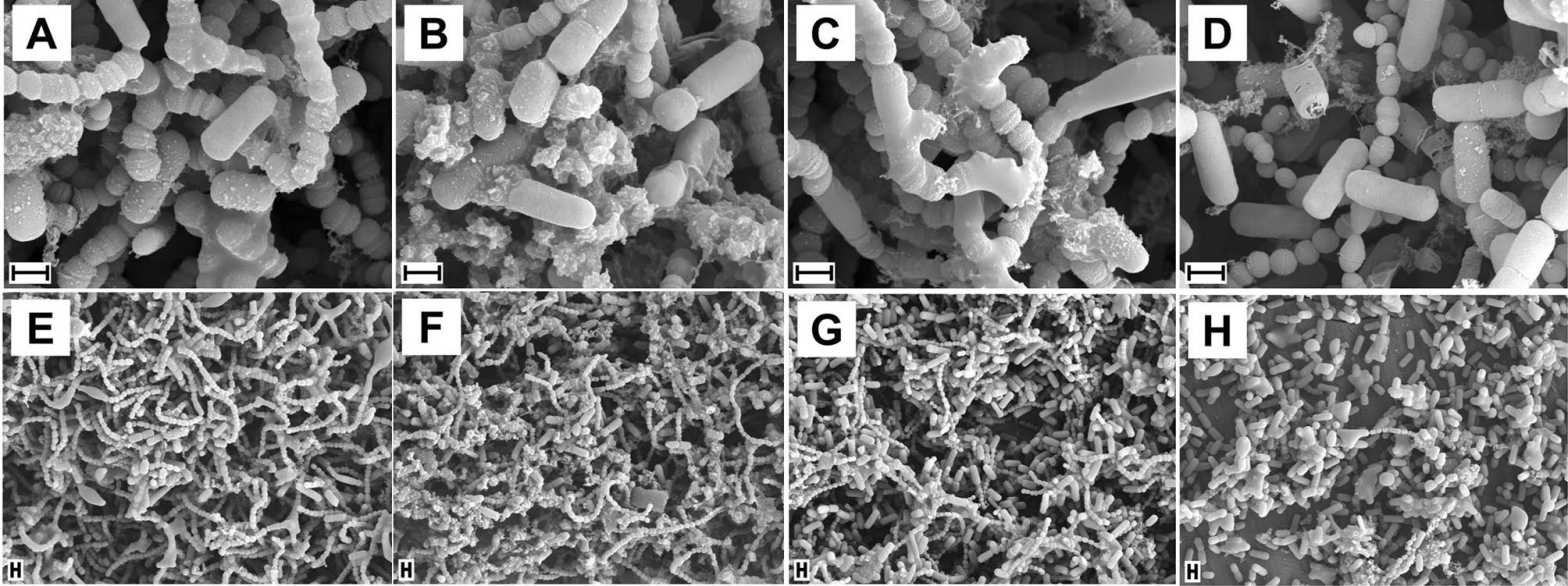
SEM images of microcosm biofilms formed on the enamel discs after 7 days according to different treatments. (**A**,**E**) Distilled water; (**B**,**F**) 50% S-PRG; (**C**,**G**) 100% S-PRG; (**D**,**H**) 0.1% CHX. Magnification: (**A**–**D**) × 10,000; (**E**–**H**) × 2,000. Scale bars: 1 μm. (n = 2).

SEM analysis was performed to observe the changes in the morphologies of the bacterial cells in the biofilm following the S-PRG treatment for 7 days (Fig. 2). S-PRG eluate groups (50% and 100%) did not show any differences in the amounts of bacteria on the surface of the specimens compared to that of the DW group. However, a few adherent bacteria were observed for the 0.1% CHX group, which shows clear membrane damage and degeneration of cells in comparison to the DW group, where only a slight change in the bacterial morphology was observed. In the SEM images of the 50% and 100% S-PRG eluate groups, no damaged bacterial cells were observed, similar to the results for the DW group.

### Biofilm thickness and live/dead cell ratio

**Figure 3:**
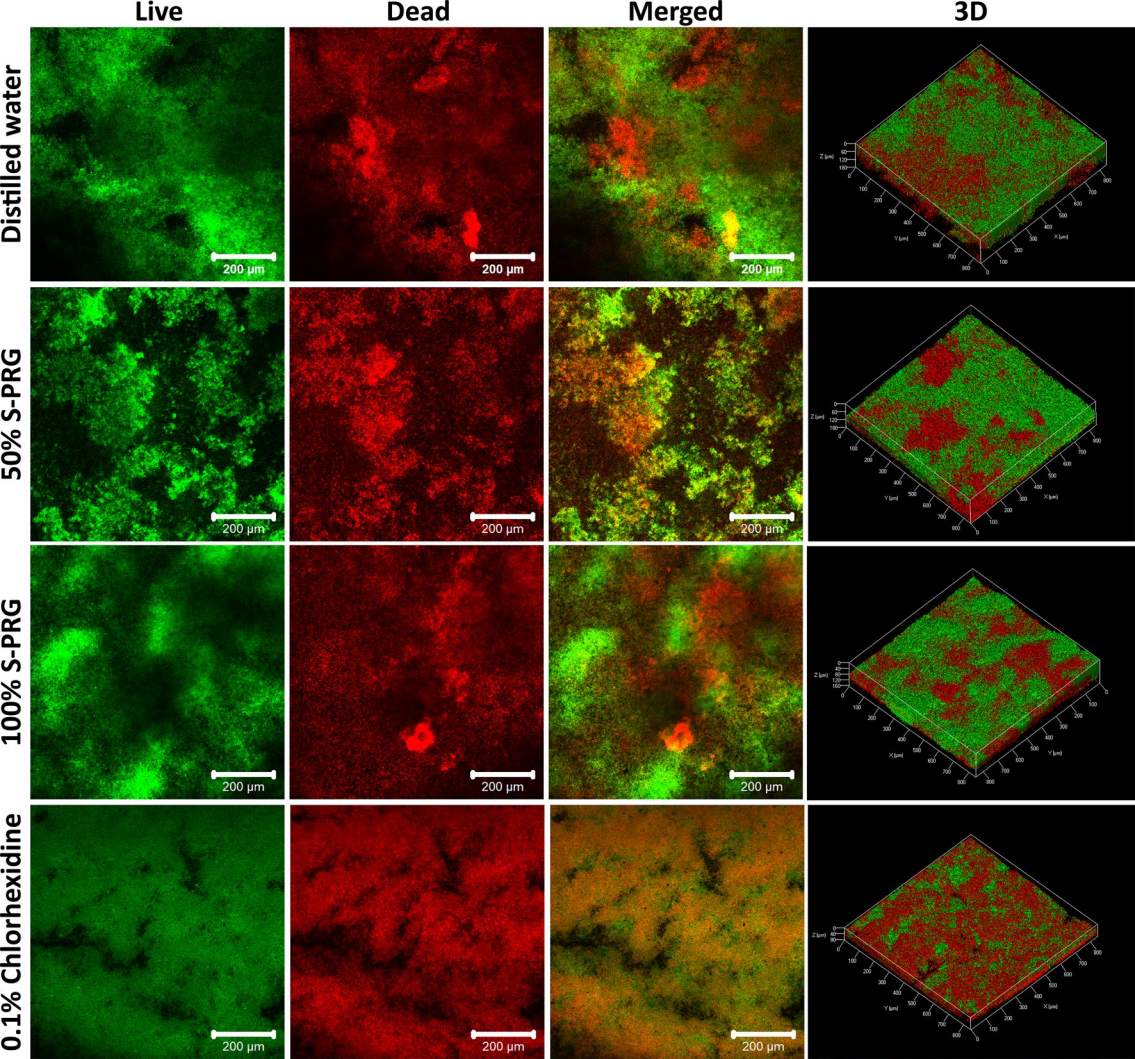
CLSM images of live/dead cells on stained microcosm biofilms after 7 days according to different treatments (n = 2).

In the CLSM images of the final mature biofilm on the seventh day (Fig. 3), the live/dead cell ratio for the DW group was 0.96 ± 0.10, which was slightly lower than the values for the S-PRG groups (50% and 100%; 1.05 ± 0.04 and 1.07 ± 0.05, respectively). The ratio for the CHX group was 0.84 ± 0.04, which was the lowest value among all groups. In addition, the biofilm thicknesses for the 50% S-PRG and 100% S-PRG groups were 101.29 ± 5.50 and 107.63 ± 16.49 μm, respectively. The DW and CHX thicknesses were 118.30 ± 53.65 and 80.21 ± 17.23 μm, respectively.

## Discussion

In this study, the antibacterial and antibiofilm effects of an S-PRG eluate were evaluated for clinical relevance by simulating the use of mouthwash (twice a day). In addition, the microcosm biofilm model, a preclinical model that simulates the real oral microbial ecosystem that considers the bacterial interactions, was used instead of real dental plaque^12^. Short-time exposure of S-PRG eluate did not show any antibacterial and anti-biofilm effects on dental biofilms. Therefore, the null hypothesis was accepted.

As a result of this study, the number of total viable bacteria and aciduric viable bacteria did not significantly decrease in the S-PRG eluate-treated group as compared to that in the negative control group. The effect of the S-PRG eluate on the growth inhibition of bacteria has been considered controversial thus far. In a previous study that reported the antibacterial effect, the addition of 25% S-PRG eluate significantly reduced the cell viability of Streptococci adjusted to 1 × 10^5^ CFU/ml; however, this reduction was not observed at 1 × 10^9^ CFU/ml^9^. In another study, the S-PRG eluate at various concentrations (0–100%) did not demonstrate the formation of an inhibition zone against saliva, revealing that the eluate alone cannot inhibit the growth of bacteria in saliva^11^. These results are consistent with this study due to the fact that the viability of bacterial cells in the microcosm biofilm did not decrease except for the CHX group. Such limited effect of the S-PRG eluate on oral bacteria can be explained by the growth characteristics of the bacteria. Bacteria growth can be inhibited by S-PRG eluate due to gene downregulation in *S. mutans* prior to the active growth phase; however, this inhibition cannot be achieved after the post-logarithmic phase^9^. In the present study, the first treatment was performed on bacteria that had been incubated for 24 h in the growth medium. It is therefore believed that the number of total and aciduric viable bacteria did not decrease significantly because bacteria of the saliva in the microcosm biofilm had already attained the post-logarithmic phase. In addition, the S-PRG eluate treatment in this study was performed for 5 min, while the S-PRG eluate and the culture medium in previous studies were incubated together for over 18 h^5,9,11^. Considering the fact that it is difficult to apply the S-PRG eluate to the oral cavity for a long time due to its liquid characteristics with flowability, the S-PRG eluate was applied to an oral microcosm biofilm for 5 min in this study; however, a significant antibacterial effect was not observed.

Similarly, the dry weight analysis did not show any significant anti-biofilm effect in the S-PRG eluate. This is believed to be due to the use of the already formed multi-species biofilm model by saliva. Nomura et al.^9^ evaluated the inhibitory effect of an S-PRG eluate for 24 h using the *S. mutans* single-biofilm model, and reported drastic reduction in the biofilm formation for 25% S-PRG eluate compared to that for 0% S-PRG eluate. In the present study, the microcosm biofilm was formed by inoculating various species from saliva. In general, multi-species biofilms are more resistant, and it is more difficult to reduce them as compared to their single-species counterparts. A multi-species biofilm composed of *S. mutans* and *Veillonella parvula* showed a stronger resistance to chlorhexidine gluconate than that demonstrated by the single-species biofilm of each strain^13^. In addition, this study evaluated the biomass in the biofilm after treating the S-PRG eluate on an already formed biofilm. Cells in biofilms exhibit a stronger drug resistance than that of planktonic cells^14^. Since the resistance of the biofilm is up to 1,000 times higher than that of the planktonic cells, a stronger antimicrobial effect is required in the biofilm. Therefore, an inhibitory effect against biofilm formation, which is one of the main effects of S-PRG, can be observed for a planktonic single bacterium; however, it cannot be observed for the already formed biofilms by multiple strains.

Some oral biofilms can be observed with red fluorescence through QLF technology^15^. The red fluorescence of the biofilm is associated with the change in the microbial composition and periodontopathic pathogens in the biofilm, and the increase in maturity and pathogenicity can be determined by dental plaque emitting the red fluorescence^16^. Previous studies have shown that red fluorescence is proportional to the degree of maturation of the biofilm and strongly correlates with the number of bacteria and enamel demineralization^17–19^. Thus, red fluorescence corresponds to the cariogenicity of the biofilm and can be useful for examining the pathogenic levels. Consistent with the prior reports, the R/G ratio in this study increased with the maturation of the biofilm^12,19^. Furthermore, in accordance with the findings of Kim et al.^18^, the R/G ratio in the present study increased considerably, exhibiting significant differences between the groups after maturation for more than three days (from the third day). These results are also consistent with previously published reports, which evaluated the effects of CHX in a microcosm biofilm model^19^. The positive control, 0.1% CHX, showed a slight increase in the R/G ratio from the third day, which is different from the results obtained for other groups. Contrarily, various concentrations of the S-PRG eluate group showed similar R/G ratios as those of the negative control group of DW. Therefore, based on the evaluations via a fluorescence reaction, we concluded that the S-PRG eluate did not inhibit the pathogenicity of the biofilms as the biofilm matured.

Although it is well-known that the ions in an S-PRG eluate are involved in various mechanisms, the antibiofilm mechanism has not yet been determined. According to previous studies, BO_3_^3−^ is known to exhibit antibacterial activity and quorum sensing inhibition^20,21^. Dembitsky et al.^22^ demonstrated the inhibitory effect of BO_3_^3–^ on quorum sensing in fungi; this indicates the role of S-PRG in inhibiting quorum sensing, which is a key component in biofilm formation. Moreover, F^–^ not only influences the growth and metabolism of intraoral bacteria, such as the inhibition of ATPase, but also binds to Sr^2+^ to form an acid-resistant layer and converts hydroxyapatite into a fluoride-apatite complex, which inhibits demineralization^23,24^. Despite of these mechanism of S-PRG, the concentration of ions contained in 100% S-PRG eluate used in this study showed no antibacterial effect. Especially BO_3_^3−^ and F^−^ was even 10 and 15 times higher than in a previous study^5^. This previous study reported antibacterial effect with a reduction from approximately 3 × 10^8^ CFU/ml to 1.5 × 10^8^ CFU/ml in single planktonic bacteria after incubation for 18 h. This reduction showed a statistically significant difference as compared to that of the negative control group; however, the effect of reducing the viable cell count was almost negligible, which was rendered less effective after treatment with planktonic bacteria for a long time. On the other hand, it was reported that no antibacterial effect was exhibited when inhibition by ion release was evaluated in a model using 100% S-PRG eluate^11^. Based on these results, S-PRG contains ions with antimicrobial and anti-biofilm effects; however, the effect of each ion may not be insufficient when treated for a short time in a multi-bacterial model.

Based on the SEM images, we confirmed that the S-PRG eluate did not damage or denature the cell membrane of the bacteria, which was consistent with the results for the negative control. In particular, it is believed that the ions in S-PRG eluate have no effect on the bacteria cell membrane in the biofilm. In contrast, the morphology of the bacteria was partially denatured after the CHX-treatment. Because of the antibacterial mechanism of CHX, where the positively charged CHX attaches to the negatively charged bacterial cell membrane resulting in damage of the cell membrane, cells were denatured^25^. In the CLSM analysis, qualitative and quantitative evaluations were performed to investigate the live/dead cells and calculate the thickness of the biofilm treated with S-PRG eluate. Further, S-PRG eluate did not show a distinct difference as compared to the biofilm treated with DW. A previous study reported that the effect of S-PRG eluate on *S. mutans* biofilm was evaluated by using CLSM, wherein 25% S-PRG eluate significantly inhibited the biofilm formation and thickness as compared to the effect of its 0% counterpart^9^. However, the biofilm was formed after incubation with S-PRG for 18 h and then evaluated. This present study tried to evaluate the possibility of using S-PRG eluate as a mouthwash. Since it is impossible to apply a liquid antibacterial agent to the oral cavity for a long time, it must be effective for a short time to apply S-PRG in the form of a rinsing liquid in daily life. In light of this view, S-PRG eluate is believed to be difficult to use in the clinical field as an antibacterial substance alone.

To exhibit a clinically significant antimicrobial effect in the oral cavity, two variables should be considered: the effective concentration of the antimicrobial and contact time. Several previous studies have evaluated an S-PRG incorporated into dental materials. In a prior investigation of the ions in the dentin, upon the application of the S-PRG sealer, ions (Al, B, F, Na, Si, Sr, and Zn) were continuously detected in the dentin for 90 days^26^. These results suggested that the S-PRG ions were continuously released, and penetrated the hard tissue for a duration of 90 days. Furthermore, Ogawa et al. found that various ions (Al, B, and Sr ions) were incorporated into the enamel in a time-dependent manner when human enamel was immersed in the S-PRG filler eluate for 1 to 4 weeks. Therefore, if the treatment of S-PRG eluate is performed for a longer time than that in the present experiment, the biofilm inhibitory effect could possibly be observed. However, unlike restorative or filling materials such as S-PRG filler, it is not possible to apply an S-PRG eluate as a mouthwash for more than 24 h. Due to these limitations, an antimicrobial effect was not observed in the microcosm biofilm when S-PRG eluate (25–100%) was applied for a short-term exposure according to the protocol of using a mouthwash. Therefore, in order to apply S-PRG eluate clinically, a modification is necessary to achieve sufficient antibacterial and anti-biofilm effects even for a short intervention.

## Materials and methods

### Preparation of S-PRG eluates

S-PRG eluates were formulated by mixing DW with S-PRG filler (Shofu Inc., Kyoto, Japan) in a 1:1 ratio (1 L: 1,000 g), followed by stirring at 23 °C for 24 h. And the mixture was centrifuged to precipitate the S-PRG filler. The supernatant solution was then filtered using a Chromato Disk (25A Hydrophilic Type, Diameter: 25 mm, pore size: 0.2 µm; GL Sciences Inc., Tokyo, Japan) to obtain the various solution concentrations (25%, 50%, and 100%)^27^. Furthermore, the elemental analyses of the BO_3_^3–^, Na^+^, Sr^2+^, Al^3+^, SiO_3_^2–^, and F^–^ ions released from the S-PRG fillers were performed using inductively coupled plasma atomic emission spectroscopy (ICPS-8000, Shimadzu Corp., Kyoto, Japan) and fluoride ion electrodes (Model 9609BNWP, Orion Research, Boston, MA, USA) (Table 2).

**Table 2:** Concentrations of ions released from S-PRG filler.

	BO_3_^3−^	Na^+^	Sr^2+^	F^−^	Al^3+^	SiO_3_^2−^
25% S-PRG	348.3	97.9	34.2	28.6	2.4	2.7
50% S-PRG	696.6	195.8	68.3	57.3	4.9	5.4
100% S-PRG	1,393.2	391.6	136.7	114.5	9.7	10.8

Concentration values are in parts per million.

### Preparation of enamel specimen

The labial surfaces of bovine incisors without cracks or white spots were sectioned (8 mm × 3 mm), embedded in a circular acrylic mold, and then ground using 600–1,000 grit abrasive paper (SiC Sand Paper, R&B Inc., Daejeon, South Korea) to obtain discs with a uniform surface. Thereafter, the enamel specimens were embedded 1 mm below the surface of the acrylic mold to create a space in which a biofilm could accumulate.

### Dental microcosm biofilm formation

Dental microcosm biofilms were formed using the model described in detail by Lee et al.^19^ Ethical approval for collecting human saliva was granted by the Ethics Committee of Yonsei Dental Hospital, Korea (IRB No. 02-2015-0051). The study was performed according to the guidelines of the Declaration of Helsinki. Saliva samples were collected from healthy adult donors who had not performed oral hygiene within 24 h, and had not taken antibiotics or exhibited any cariogenic activity or periodontal disease within the last three months. Informed written consent was obtained from the donors. The collected saliva samples were filtered through sterilized glass wool (Duksan Pure Chemicals Co., Ltd., Ansan, Korea) and then diluted in sterile glycerol (Duksan Pure Chemicals Co., Ltd., Ansan, Korea) to prepare stock solutions with a final concentration of 30%. The stock solutions were stored at − 80 °C, and the same batch of frozen saliva was used as an inoculum for each set of experiments. The stock saliva (1.5 ml) was inoculated with each of the specimens in a 24-well cell plate and incubated at 37 °C and 10% CO_2_ for 4 h. The specimens were transferred to new wells with a basal medium mucin growth medium mixed with 0.3% sucrose and incubated in an anaerobic environment (80% N_2_, 10% CO_2_, and 10% H_2_) at 37 °C for 7 days to form microcosm biofilms.

### Treatment

To simulate the use of the S-PRG eluate as an oral rinse, the treatment procedure was performed twice a day for 5 min repeatedly. Biofilms formed on the enamel specimens for 4 h were immersed in 1.5 ml of 25%, 50%, and 100% S-PRG eluate, 0.1% CHX (positive control), and DW (negative control) for five minutes in each treatment. After the treatment, the specimens were washed three times with cysteine peptone water (CPW) to remove the excess treatment solution. After each treatment, the specimens were returned to the growth medium.

### Microbial composition

To compare the antibacterial effects of S-PRG eluate at various concentrations, the total and aciduric bacterial counts in the biofilms formed during the 7 days of treatment were measured. This was achieved by first washing the enamel specimens with 1.5 ml of CPW to remove the planktonic bacteria and then immersing the specimens in a conical tube containing 2 ml of CPW. Biofilm bacteria were then suspended by sonication and vortexing for one minute each, and the bacterial suspension was serially diluted and spread on a 5%-tryptic soy broth agar plate (which is a selective medium) and a brain heart infusion agar plate adjusted to pH 4.8. The plates were incubated at 37 °C for 72 h in anaerobic conditions to yield the total and aciduric bacterial counts, which were quantified in colony-forming units (CFUs). The number of enamel specimens in each group was 13; this number was used for measuring the bacterial counts.

### Dry-weight analysis

To quantitatively determine the biomass of the biofilms, dry weight analysis was performed using the method reported by Lemos et al.^28^ with minor modifications. Briefly, the biofilms that matured on the specimens for 7 days were washed three times with 1.5 ml of CPW and transferred to sterile tubes containing 2 ml of CPW. Then each tube was placed in an ultrasonic bath for 10 min to detach the biofilm formed on the specimen. Then, the specimen was removed from the tube and centrifuged at 5,500×*g* for 10 min at 4 °C. After carefully discarding the supernatant, 2 ml of DW was added and vortexed to resuspension. The process of discarding the supernatant after centrifugation under the same conditions was repeated. A pellet resulting from this process was used to measure the dry weight of 1 ml after resuspension in 2-ml DW. This analysis was repeated 3 times, resulting in a total of 9 samples in each group.

### Optical quantification of red fluorescence emitted from biofilm

A quantitative light-induced fluorescence-digital (QLF-D) Biluminator device (Inspektor Research Systems, Amsterdam, The Netherlands), which comprised a digital SLR camera with QLF technology, was used to evaluate the cariogenicity of the biofilms based on their autofluorescence responses. To evaluate the red fluorescence intensity of the biofilms using QLF technology, which is an indicator of cariogenicity, the biofilms formed on all specimens were imaged using the following settings: shutter speed of 1/45 s, aperture of 3.2, and ISO speed of 1,600. To compare the red fluorescence intensities between the treatment groups, a region of interest in the biofilm was selected in the fluorescence images^17^. The red, green, and blue fluorescence values for all pixels in the corresponding region were measured such that the R/G ratio could be computed using an image analysis program (ImageJ with 64-bit Java 1.8.0_112 National Institute of Health, USA). Thirteen specimens in each group were used for this optical quantification.

### Scanning electric microscopy (SEM) analysis

SEM was used to investigate the morphological changes in the cariogenic biofilms after treatment with the S-PRG eluate at different concentrations. After the completion of the treatment and biofilm formation for 7 days, the enamel specimens were prefixed with 2% glutaraldehyde for at least 6 h, washed with 0.1 M phosphate-buffered saline for two hours, and postfixed with 1% osmium tetroxide. The postfixed enamel specimens were dehydrated using a graded series of ethanol solutions (50%, 60%, 70%, 80%, 90%, and 95%), subjected to critical-point drying (HCP-2, Hitachi, Ltd., Tokyo, Japan), and coated with gold using an ion sputter coater at 6 mA for 6 min. The surface of the biofilm was visualized and photographed at magnifications ranging from 10,000 × to 2,000 × by SEM (FE SEM S-800, Hitachi, Ltd., Tokyo, Japan) at an acceleration voltage of 20 kV. Two specimens in each group were used for the SEM analysis.

### Confocal laser scanning microscopy (CLSM) analysis

Structural and quantitative analyses of the biofilm were performed using CLSM (LSM880, Carl Zeiss, Jena, Germany) equipment. The biofilm that formed over 7 days of treatment was stained using the LIVE/DEAD BacLight Bacterial Viability Kit (Molecular Probes, Eugene, OR, USA) according to the manufacturer’s instructions. Live bacterial cells were stained with SYTO9 (with excitation and emission maxima at 480 and 500 nm, respectively) and exhibited green fluorescence, while dead bacterial cells were stained with propidium iodide (with excitation and emission maxima at 490 and 635 nm, respectively) and exhibited red fluorescence. The biofilm was visualized at five randomly assigned positions using CLSM. Axially stacked biofilm images were captured by the Zen software (ZEN 2.6 blue edition, Carl Zeiss Microscopy GmbH, 2018), and the COMSTAT plug-in (Lyngby, Denmark) in ImageJ software was used to quantify the biomass and live/dead cell ratio of the biofilm. Two samples in each group were used for the CLSM analysis.

### Statistical analysis

G Power software (Version 3.1, University of Dusseldorf, Dusseldorf, Germany) was used to calculate the required sample size. Based on the statistical power, alpha level, and effect size of 0.80, 0.05, and 0.25, respectively, a minimal sample size of 9 specimens was used for each group. Cell viability, biomass, and R/G values were evaluated using the Mann–Whitney U-test complemented by the nonparametric Kruskal–Wallis test to evaluate the significance of antibiofilm effects of the S-PRG eluate at different concentrations on the microcosm biofilm. All data were analyzed using PASW Statistics 18.0 (IBM SPSS Statistics 20.0 for Windows, SPSS, Chicago, IL, USA) software with a significance cutoff of 0.05.
